# A Triple Co-Delivery Liposomal Carrier That Enhances Apoptosis via an Intrinsic Pathway in Melanoma Cells

**DOI:** 10.3390/cancers11121982

**Published:** 2019-12-09

**Authors:** Nina Filipczak, Anna Jaromin, Adriana Piwoni, Mohamed Mahmud, Can Sarisozen, Vladimir Torchilin, Jerzy Gubernator

**Affiliations:** 1 Department of Lipids and Liposomes, Faculty of Biotechnology, University of Wroclaw, 50-383 Wroclaw, Poland; anna.jaromin@uwr.edu.pl (A.J.); adriana.piwoni@gmail.com (A.P.); mohamedzwawi@yahoo.com (M.M.); jerzy.gubernator@uwr.edu.pl (J.G.); 2 Department of Food Science and Technology, Faculty of Agriculture, University of Misurata, Misurata 2478, Libya; 3 Center for Pharmaceutical Biotechnology and Nanomedicine, Northeastern University, Boston, MA 02115, USA; cansarisozen@gmail.com (C.S.); v.torchilin@northeastern.edu (V.T.); 4 Department of Oncology, Radiotherapy and Plastic Surgery I.M. Sechenov First Moscow State Medical University (Sechenov University), 119991 Moscow, Russia

**Keywords:** anacardic acid, mitoxantrone, targeted drug delivery, liposomes, melanoma, apoptosis, ascorbic acid

## Abstract

The effectiveness of existing anti-cancer therapies is based mainly on the stimulation of apoptosis of cancer cells. Most of the existing therapies are somewhat toxic to normal cells. Therefore, the quest for nontoxic, cancer-specific therapies remains. We have demonstrated the ability of liposomes containing anacardic acid, mitoxantrone and ammonium ascorbate to induce the mitochondrial pathway of apoptosis via reactive oxygen species (ROS) production by the killing of cancer cells in monolayer culture and shown its specificity towards melanoma cells. Liposomes were prepared by a lipid hydration, freeze-and-thaw (FAT) procedure and extrusion through polycarbonate filters, a remote loading method was used for dug encapsulation. Following characterization, hemolytic activity, cytotoxicity and apoptosis inducing effects of loaded nanoparticles were investigated. To identify the anticancer activity mechanism of these liposomes, ROS level and caspase 9 activity were measured by fluorescence and by chemiluminescence respectively. We have demonstrated that the developed liposomal formulations produced a high ROS level, enhanced apoptosis and cell death in melanoma cells, but not in normal cells. The proposed mechanism of the cytotoxic action of these liposomes involved specific generation of free radicals by the iron ions mechanism.

## 1. Introduction

Melanoma, one of the most aggressive types of cancer, arises from the transformation of normal melanocytes. This type of cancer is characterized by uncontrolled divisions, dysregulation of cell processes and the ability to invade and create metastases even at an early stage of development [1,2].

The basic method of treating melanoma is the surgical removal of the neoplastic lesions with a margin of healthy tissue. In addition, chemotherapy, radiotherapy and immunotherapy are also used. However, despite aggressive treatment, complete remission is often not observed. The prognosis depends on the depth of tumor cell infiltration and the clinical stage of the cancer. In early stages (infiltration depth up to 1 mm), a cure can be up to 90–100% and decreases as the cancer progresses. Conventional chemotherapy with dacarbazine and temozolomide has few side effects, but it is not very effective. Interleukin-2 and other immunocytokines have been known to be effective in melanoma treatment, but their use is limited due to high systemic toxicity [3,4,5], which is why using this method of treatment is still problematic [6]. Another approach to melanoma treatment is to inhibit negative regulation by binding to CTLA-4. CTLA-4 blocking promotes stimulation of adaptive immunity and T cell activation. CTLA-4 blocking antibodies have shown efficacy in various mouse models of malignancy when administered alone or in combinations with vaccines, chemotherapy and radiation. Some of anti-CTLA-4 antibodies were recently developed, including ipilimumab and tremelimumab. Both of the letter antibodies have been extensively evaluated for melanoma; in particular, ipilimumab has recently been approved as second line monotherapy in the treatment of advanced melanoma. Tremelimumab is currently being evaluated in Phase II trials as monotherapy for melanoma [7,8], however standards of melanoma treatment are dacarbazine and temozolomide [8]. Other co-inhibitory pathway uses the programmed cell death receptor 1 (PD-1), which is an inhibitory receptor present on activated T cells. When PD-1 binds to its ligand (PD-L1), which is often expressed on activated cancer cells, the ability to T cells to produce an effective immune response is reduced. Antibodies directed against PD-1 (nivolumab, pembrolizumab) or PD-L1 may therefore help to restore or enhance the anti-tumor immune response and induce tumor remission in patients with advanced melanoma. Although this approach has a significant impact on survival, half of the patients do not respond well to treatment. Possible mechanisms of poor response to treatment are numerous and may include the following: (1) no preexisting anti-tumor immune response, (2) intratumoral development of immunosuppressive molecules such as indoleamine dioxygenase (3) and oncogenic pathways of melanoma, which activate immunosuppressive programs such as WNT/β-catenin. Therefore, more research is needed to enable appropriate selection of patients who are likely to benefit from this approach [9,10].

These results may be improved by using combination therapy. In this report, the main chemotherapeutic was mitoxantrone, used in combination with two additional active agents. Mitoxantrone (MIT) [11] has been used extensively in the treatment of cancers such as acute myeloid leukemia, non-Hodgkin’s lymphoma, prostate cancer and breast cancer as well as in active forms of progressive multiple sclerosis [12,13]. The antitumor effect of MIT is based on its ability to interact with DNA, where it forms a covalent complex with topoisomerase II, which prevents the rejoining of the single DNA strand during replication, and consequently inhibits DNA replication and RNA transcription [14,15,16], and the cell cycle at various stages [17]. Despite its wide application, MIT can have many side effects including cardiotoxicity [18,19]. Recently, the use of natural dietary antioxidants has been considered for use, to minimize cytotoxicity and tissue damage with anti-cancer agents [20]. Some natural substances, such as ascorbic acid, improve anti-cancer activity of some chemotherapeutic drugs [21]. Ascorbic acid is a water-soluble antioxidant as well as an enzyme co-factor produced by plants and certain animals. It is a powerful reducing agent that effectively neutralizes potentially harmful free radicals generated as a result of metabolic processes [22,23,24]. In contrast to normal cells, ascorbic acid becomes more toxic towards cancer cells because of the increased uptake of its oxidized form, dehydroascorbate (DHA), via the GLUT1 glucose transporter. Release of iron ions from the cytoplasmic iron storage protein, ferritin (iron ions are much more abundant in cancer cells due to transferrin receptors overexpression), which become available for DHA, results in production of hydrogen peroxide and depletion of the reduced glutathione pool by a cyclic oxidation-reduction mechanism [25]. Several studies have shown a positive effect of ascorbic acid in the treatment of cancer and a significant reduction in chemotherapy-related adverse reactions in patients [26].

Iron can be released from ferritin by additional biological reductants including thiols and reduced flavins [27]. One of the anticancer activities of the anthracyclines and its derivatives is free radical formation by reaction with iron ions [28,29,30]. Therefore, it appeared reasonable to use the ammonium ascorbate ion gradient for the encapsulation of the anthracycline derivative, MIT, to maximize its anticancer potential. The iron-based free radical mechanism for MIT action is increased in cancer cells known to accumulate iron [31]. 

In this work, we tested liposomes for the ascorbic acid effect alone or combined with MIT or with anacardic acid (AA), which is another natural compound with potential anticancer activity. Anacardic acid inhibits histone acetyltransferases (HAT) activity of p300 and P300/CBP-associated factor (PCAF) in vitro [32]. Blocking the p300 protein inhibits the NF-κB pathway regulating cell proliferation, survival and inflammation [33]. Anacardic acid may also reduce VEGF-dependent angiogenesis, which can lead to inhibition of tumor growth [34]. Its activity has been demonstrated with melanoma cells [35], colon, breast, lung, cervical and renal cancers [36]. It may also affect the growth of hormone-dependent tumors by inhibiting the activity of androgen and estrogen receptors [37,38]. However, the exact mechanism of anticancer activity of anacardic acid is not fully understood. It is believed to be involved in the modulation of the expression of several genes involved in the cell cycle and apoptosis. The action of anacardic acid causes the arrest of the cell cycle in the S phase and the induction of apoptosis, most probably by reducing the expression of genes including Bcl-2 and Bax [39]. Remarkably, the hydrogenation of anacardic acid to form its saturated acid did not alter the inhibitory activity, thus eliminating the importance of saturation status in the mechanism of inhibition. [32]. Tested liposomal formulations containing 5 mol% of the AA with MTX encapsulated by means of an ammonium ascorbate gradient exhibited remarkably high toxicity toward both used melanoma cancer lines, but not to non-cancer NHDF cells, where the protective influence of ascorbate was observed.The main purpose of this research was to develop liposomes co-loaded with anacardic acid, mitoxantrone and ammonium ascorbate, and investigate the combined effects of these liposomal formulations on human melanoma cancer cells.

## 2. Results

### 2.1. Liposomes Preparation and Characterization

**Figure 1:**
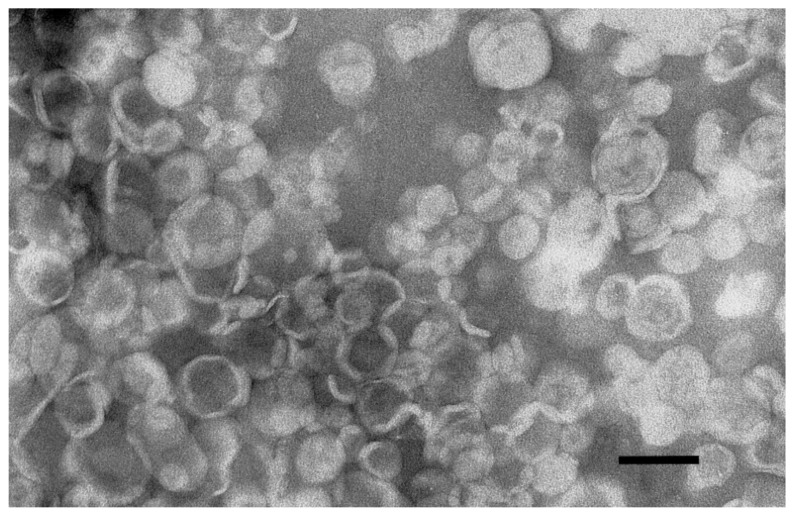
Transmission electron microscopy image of 5 mol% AA-incorporated liposomes. Scale bar = 100 nm.

As shown in Table 1A, liposomes containing different amounts of anacardic acid had an average size around 110 nm with a narrow size distribution and slightly negative charge. Additionally, morphological analysis determined by TEM microscopy confirmed the presence of circular structures with uniform size in AA-containing liposome samples (Figure 1).

In all cases MIT encapsulation was very efficient (above 98%). All of these parameters remained nearly the same (Table 1B) after targeting liposomes with transferrin.

### 2.2. Liposomes Demonstrate Selectivity toward Melanoma Cells and Low Cardio- and Hepatotoxicity and a Lower Hemolysis Ratio

The cytotoxicity of the free drug and liposomal formulations was tested on two human melanoma cell lines A375 and Hs294T. Mitoxantrone was entrapped in liposomes using two different gradients: ammonium ascorbate (Vit C) and ammonium sulphate (AS). Liposomes enriched with anacardic acid (AA) and liposomes without AA were investigated. Cell viability was measured by MTT assay after 48 or 72 hrs from the addition of free drug or liposomes and the cell survival curves were determined.

Both cell lines were sensitive to MIT in a dose-dependent manner. Empty AA liposomes with Vit. C showed only slight toxicity to melanoma cells, which was approximately the same in both cell lines. This toxicity was directly proportional to the amount of anacardic acid. This effect was much less visible for liposomes with ammonium sulphate. The presence of AA significantly increased the cytotoxicity of the liposomal MIT, both with liposomes containing ammonium ascorbate and for liposomes with ammonium sulphate. An additional significant cytotoxic effect of ascorbic acid was observed at higher concentration and after a longer incubation time (72 h), especially in the A375 cell line. The difference between Lip AA5 MIT Vit. C and Lip AA5 MIT AS was significant over the entire range of concentrations tested. The patterns observed after 48 hours of incubation remain practically unchanged when incubated for 72 hours.

The NHDF skin fibroblast cell line was used as a normal control for melanoma cells to check specificity of the action of anacardic acid and mitoxantrone, because the highly desirable feature of the drug carrier is its low toxicity against cells that are not cancerous.

A toxic effect of anacardic acid on normal cells, above 5 mol% concentration for formulations containing ammonium ascorbate was observed. However, a formulation containing 5 mol% of anacardic acid and ammonium ascorbate was not toxic to the normal skin cells. 

Based on the survival curves for all formulations, the IC_50_ parameter was determined (Table 2). The plain liposomes were characterized by an IC_50_ concentration ranging from several to several hundred μM of total mol amount of lipids that form liposomes. In addition, anacardic acid in combination with ammonium ascorbate is more toxic to melanoma cells than in combination with ammonium sulfate (IC_50_ for Lip AA5 Vit. C. was 12.1 μM and for Lip AA5 AS was 35.8 μM for A375 after 48 h incubation). This tendency was evident for both melanoma cell lines and independent of incubation time. The reverse is true for the NHDF cell line, where the formulation with anacardic acid in combination with ammonium ascorbate is less toxic than the formulation of anacardic acid with a pH gradient generated by ammonium sulphate (IC_50_ value is more than three times higher for LipAA5 Vit.C for 48 h incubation and 138 times higher for 72 h).

For mitoxantrone-containing liposomes, IC_50_ values ranged from 0.02 to 75.05 μM (Table 2). There was also a protective effect of vitamin C in combination with anacardic acid for normal cell lines, as was the case with liposomes without a drug. Most current cancer therapies combine various therapeutic agents. 

To determine the kind of interaction occurs between the anacardic acid and mitoxantrone, the CI was determined (Table 3). By comparing the combination index values, mitoxantrone and anacardic acid were clearly shown to act synergistically or additively on melanoma cells in the presence of ammonium sulphate or ammonium ascorbate. In the presence of vitamin c, AA and MIT acted antagonistically in normal cells by contrast to ammonium sulphate. These results suggest a different molecular mechanism of action in cancer cells and normal cells. The factor limiting the use of mitoxantrone in anticancer therapy is its high cardiotoxicity. In addition, this drug undergoes transformation in the liver, which leads to hepatotoxicity. 

**Figure 2:**
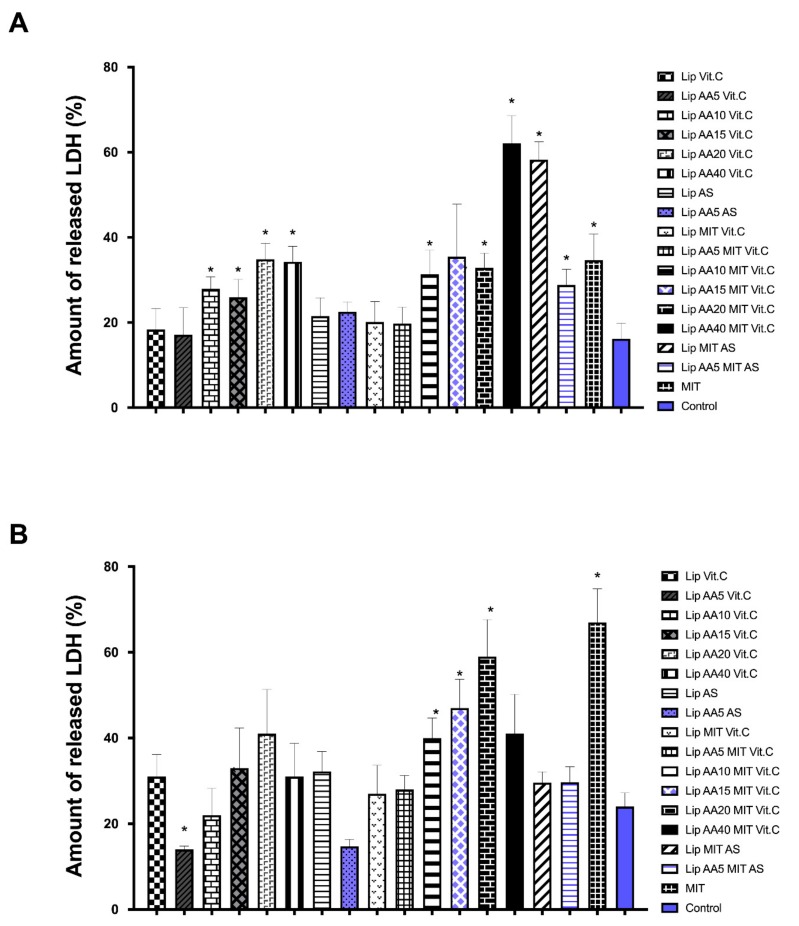
LDH released into the medium as a marker of cytotoxicity of liposomal formulation at a concentration of 10μM of MIT for line H9C2 (**A**), line H9C2 Hep-G2 (**B**). * the difference statistically significant to the control (T test) *p* < 0.05.

**Figure 3:**
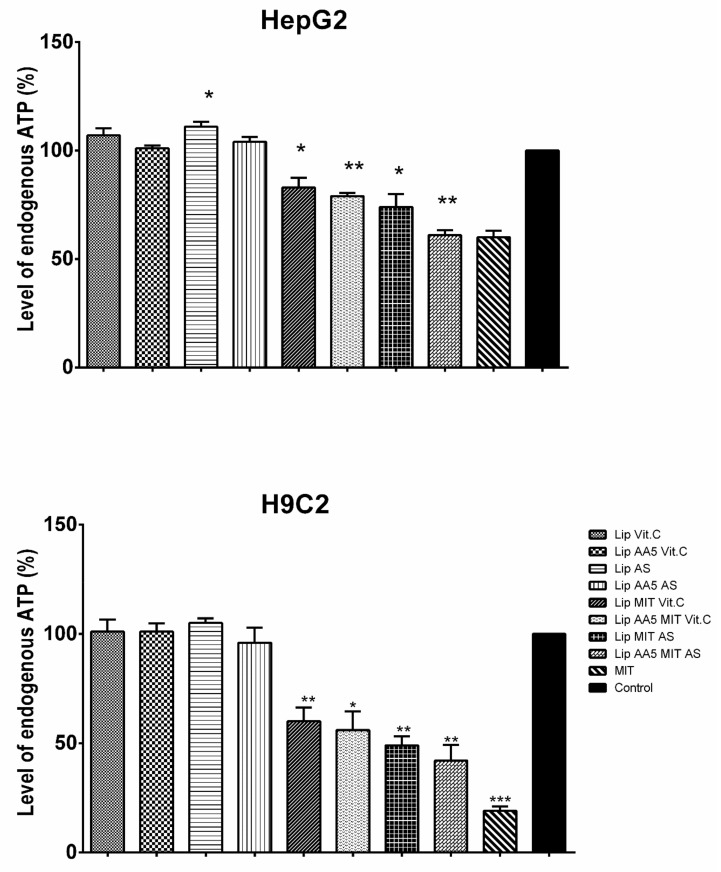
Intracellular ATP level in the Hep-G2 line (upper), line H9C2 (lower). * the difference statistically significant to the control (*T* test) *p* < 0.05; ** the difference statistically significant to the control (*T* test) *p* < 0.01; *** the difference statistically significant to the control (*T* test) *p* < 0.001.

The use of the lipid carrier was aimed to reduce of the side effects of mitoxantrone. The released LDH (Figure 2) and intracellular ATP (Figure 3) level were used to estimate hepatotoxicity and cardiotoxicity, respectively.

The results obtained on the Hep-G2 liver cell line and H9C2 rat cardiomyocytes indicate a reduction in the toxicity of mitoxantrone in the liposomal form in relation to free drug for Hep-G2 cells. In addition, the formulation anacardic acid-enriched showed no increased toxicity to liver cells, even when combined with mitoxantrone. A similar effect was obtained for H9C2 myocardial cells, except for the formulation containing 40 mol% AA and MIT, and MIT formulations with AS, which were more toxic than free drug. The higher toxicity of the latter formulations suggests the involvement of vitamin C in the protection of cells against drug toxicity. The Lip MIT AS liposomes compared to Lip AA5 MIT AS liposomes showed a noticeable reduction in the toxicity in the presence of anacardic acid. The addition of anacardic acid to the liposome membrane did not change the level of intracellular ATP for either cell line (Figure 2B). Mitoxantrone significantly reduced ATP level (up to 60% for myocardial cells), but this effect is not observed in combination with anacardic acid and ammonium ascorbate. MIT in the presence of AA and ammonium sulfate induced a much stronger cell response. In addition, MIT’s influence on the level of ATP in liver cells is smaller than in the myocardial cells. This is opposite the effect in the case of LDH, which suggests that the toxicity of mitoxantrone in HeP-G2 cells is manifested by the release of LDH, while for H9C2 cells, by the reduction in ATP levels.

**Figure 4:**
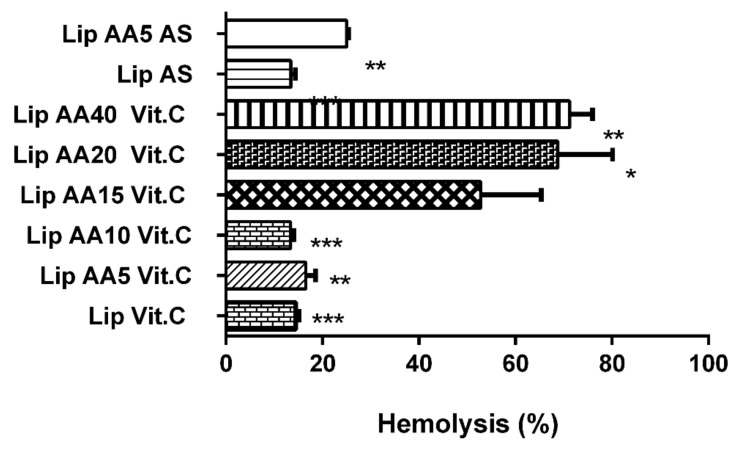
Hemolysis of human erythrocytes after incubation with liposome formulations (*T* test) * *p* = 0.0176; ** *p* = 0.0058; *** *p* = 0.0008.

The hemolytic potential of free AA and AA-enriched liposomes without drug after incubation with human erythrocytes was observed (Figure 4). Formulations were characterized by their ability to induce the release of hemoglobin from red blood cells. 

Free AA at the concentration corresponding to 5 mol% caused 40.9% of hemolysis. Values obtained for Lip AA5 Vit. C and Lip AA5 AS 16.5 and 25%, respectively suggest a protective effect after its incorporation. It is worth noting that the free form of anacardic acid in concentrations equivalent to their content in liposomes 10 mol% or more is responsible for complete membrane damage under the conditions used. Therefore, the results obtained for Lip AA10 Vit. C are extremely interesting. The hemolysis determined was at the level of 13.4%, similar to the case of control compositions without AA (Lip Vit. C and Lip AS). This observation might indicate that AA located in the membrane probably has no direct contact with erythrocytes. Unfortunately, as the fraction of this compound increases in the remaining formulations (15, 20 and 40 mol%), the protective effect becomes weaker, probably due to presence of interactions with red blood cells. Summarizing, these results demonstrate that AA-incorporated liposomes are likely to cause less toxicity than free AA after intravenous administration and support the development of formulations for in vivo administration.

### 2.3. ROS Formation Induced by Liposome Formulations

**Figure 5:**
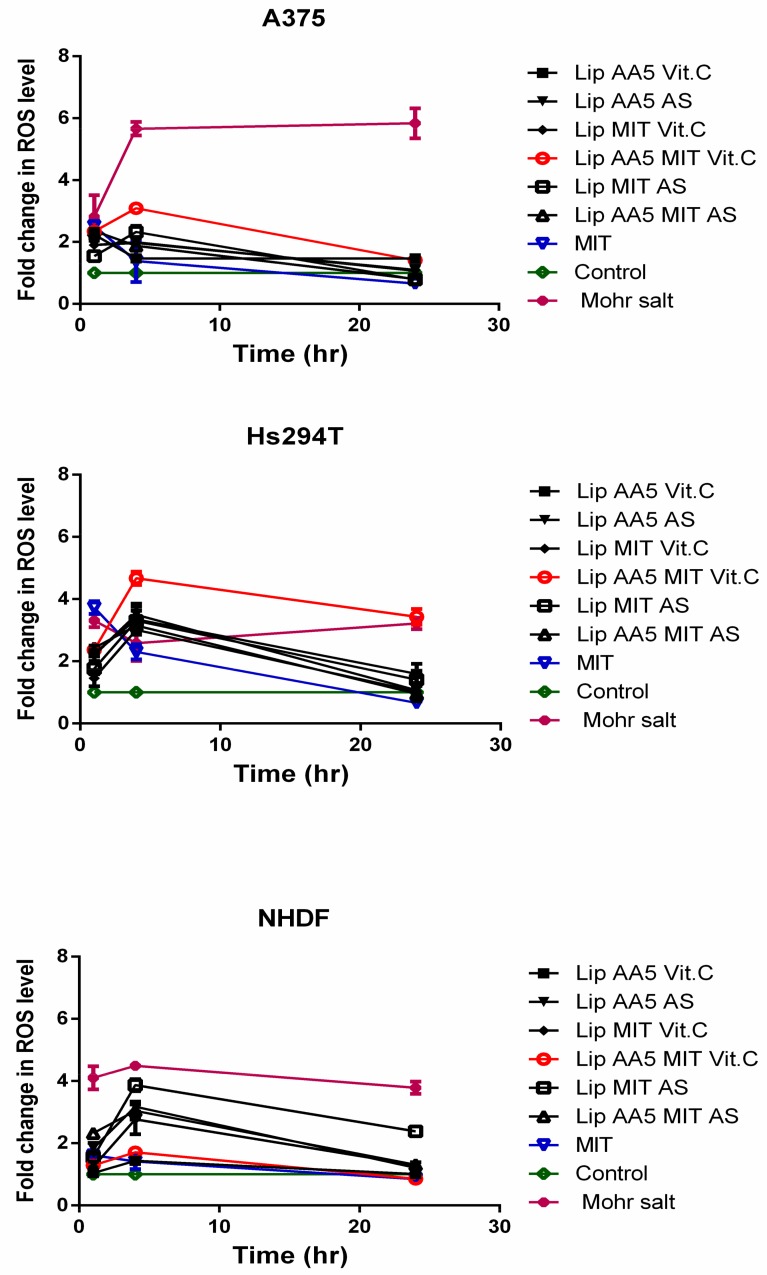
Reactive oxygen species levels in A375, Hs294T and NHDF cells after incubation with liposome formulations and free drug. The untreated cells (control) are considered as 100% of the endogenous ROS level. The Mohr salt was used as a positive control.

A possible mechanism for caspase pathway activation is the excessive production of reactive oxygen species in response to cell treatment with liposomes. The highest increase in the level of reactive oxygen species was observed 4 h after addition of liposomes (Figure 5). 

The formulation that most effectively raised the level of ROS in the cells of both melanoma lines was Lip AA5 MIT Vit. C, while the level of ROS induced by this formulation in NHDF cells was comparable to untreated cells. The time at which the level of ROS increase come before the increase in caspase activity, suggesting that increased ROS production was the cause of melanoma cell apoptosis.

### 2.4. Liposome Treatment Enhances the Apoptosis

**Figure 6:**
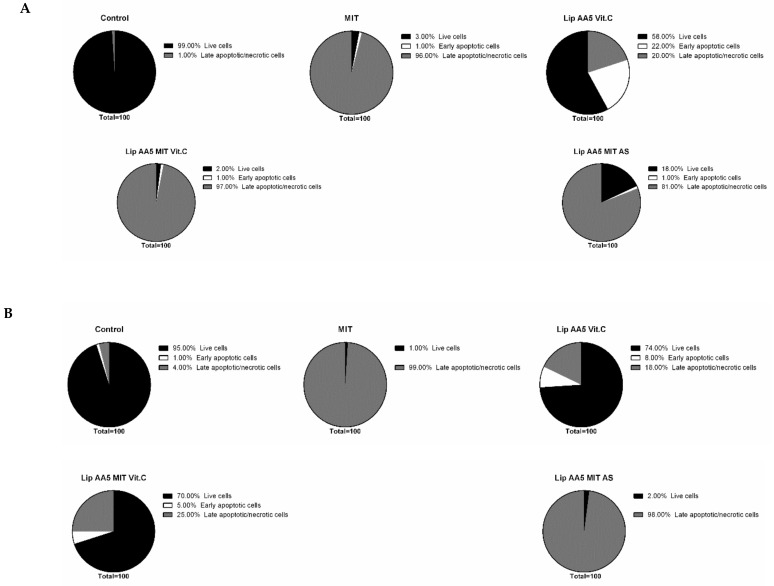
Percentage of the population of dead, living and early apoptotic (**A**) A375 and (**B**) NHDF cells after treatment with liposomes or free drug.

Using flow cytometry, the progress of apoptosis in melanoma cells after 24 hours of incubation with liposomes and free drug at a dose corresponding to the IC_50_ (48 h) was analyzed. The controls were cells not treated with liposomes. After incubations, cells were labeled with Annexin V fluorescein-quenched and propidium iodide. In early apoptosis, phosphatidylserine dislocates from the inner side of the cell membrane to the exterior and can then bind to annexin V. Propidium iodide makes it possible to differentiate the population of cells with disturbances in the cell membrane integrity. Mitoxantrone in free form reduced the population of healthy cells in favor of a late-apoptotic form, regardless of the cellular model. For A375 melanoma cells (Figure 6), the liposomal form of mitoxantrone, anacardic acid and vitamin C worked in an almost identical manner (97% of cells were in the late stage of apoptosis or necrosis). 

A similar effect was also observed with liposomal forms of anacardic acid, mitoxantrone and ammonium sulphate and formulations containing no anacardic acid. For the metastatic model of melanoma (Hs294T), the effect of free drug was comparable with Lip AA5 Vit C, while the change from vitamin C to ammonium sulfate no longer increased the population of late apoptotic or necrotic cells. This observation suggests the interaction of vitamin C, AA and mitoxantrone in the induction of melanoma cell death. It is noteworthy that the formulation of Lip AA5 Vit C was not toxic to normal skin cells (about 70% of cells remained intact). 

**Figure 7:**
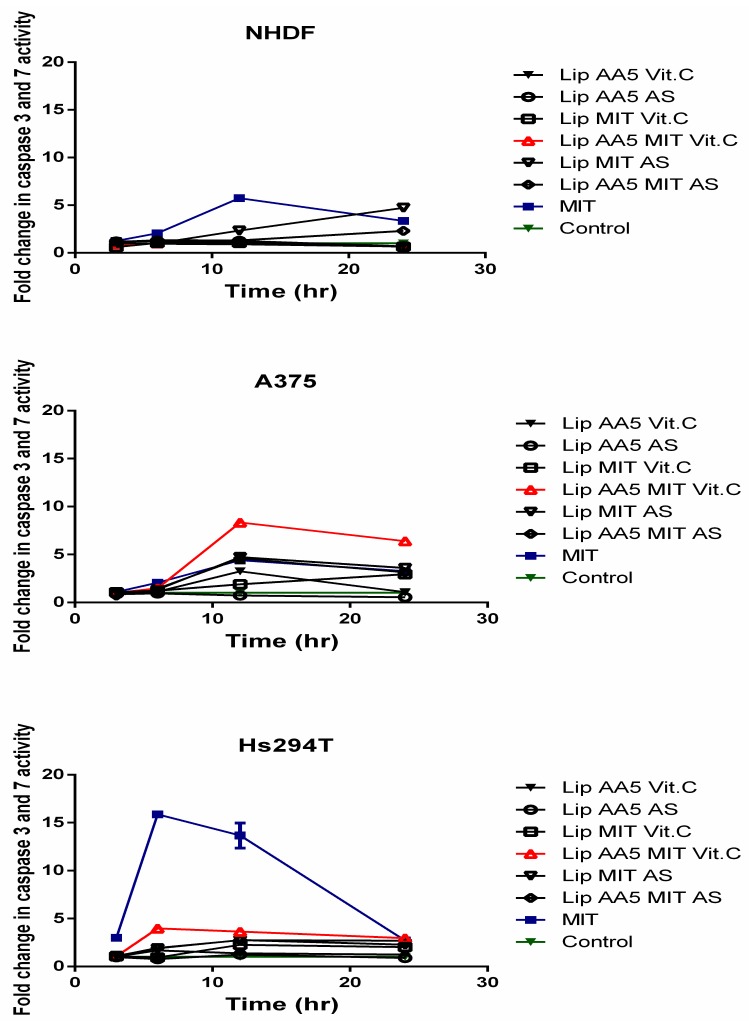
Activity of executive caspases within a 24-hour period after administration of liposomes or free drug. Caspase activity level was assumed to be 100% in cells not treated with liposomes.

This observation demonstrates the high efficacy and specificity of the developed formulation. The results indicate that cells treated with liposomes can die by apoptosis. The Caspase3/7 Glo^®^ test was performed to check whether it was caspase-dependent apoptosis. A noticeable increase in caspase activity can be observed as early as the 6 h after treatment of cells with liposomal preparations (Figure 7). 

**Figure 8:**
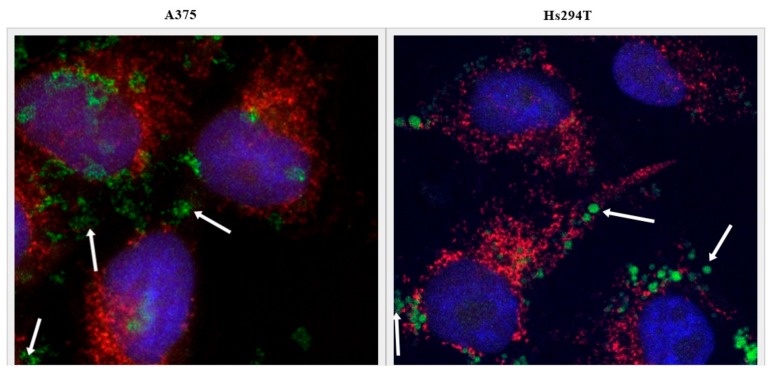
Microscopic photo of melanoma cells after treatment with liposomes (Lip AA5 MIT Vit.C). The cell nuclei are marked in blue, the red color indicates mitochondria, and the green color shows cytochrome c. The arrows indicate cytochrome c released from the mitochondria.

The highest caspase activity was observed for the Hs294T line in response to the free drug and the Lip AA5 MIT Vit C. This formulation led to the highest increase in caspase activity (eight-fold) for A375 melanoma, but only 12 hr after administration of liposomes. Lip AA5 MIT Vit C did not elicit any response in NHDF normal cells, but the free drug and the ammonium sulfate-containing formulation caused an increase in caspase activity, after 24 hr of incubation. The results obtained indicate caspase-dependent apoptosis in response to treatment with liposome formulations, and that Lip AA5 MIT Vit C formulation selectively induced caspase-dependent apoptosis in melanoma cells. To check which apoptosis pathway is promoted cytochrome C released study was performed. Cytochrome C appearance outside of mitochondria in treated with liposomes cells suggested activation of mitochondrial pathway of cell apoptosis (Figure 8). 

**Figure 9:**
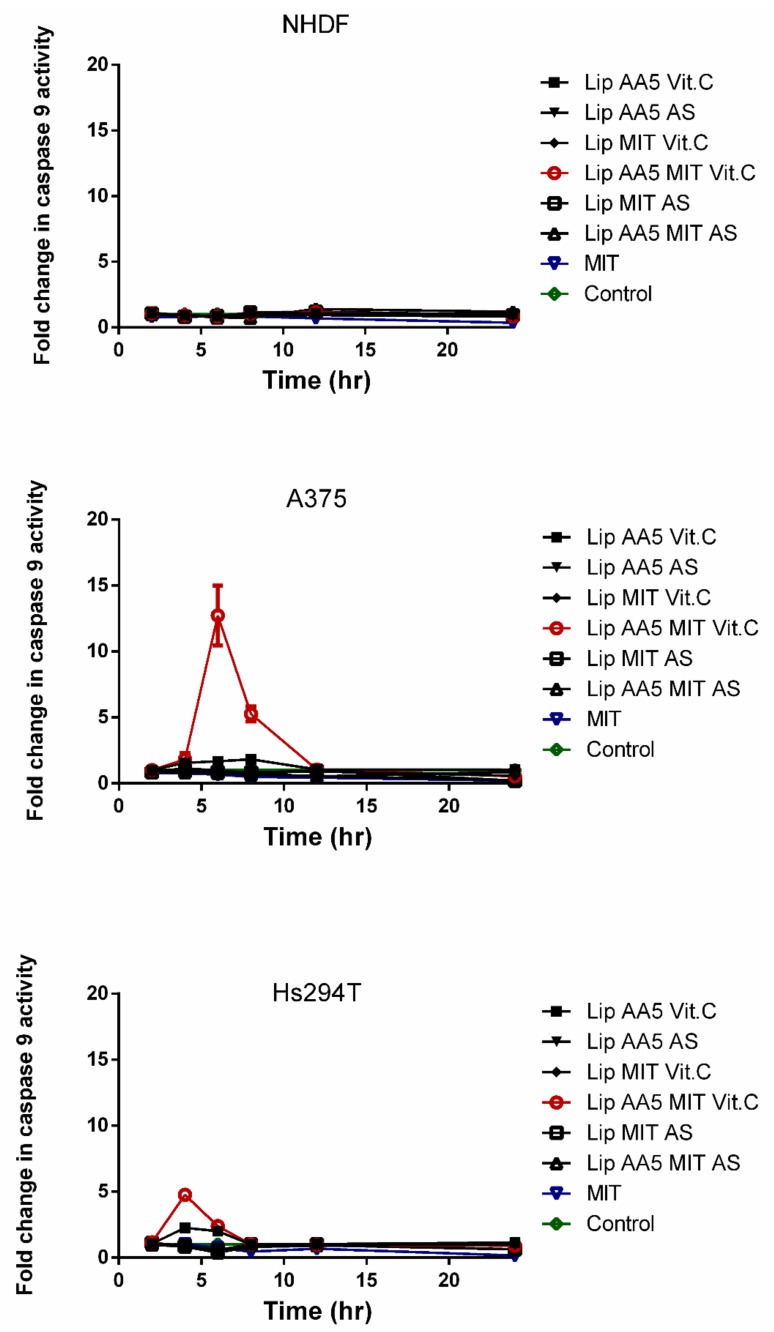
Caspase 9 activity in a 24-hour period after administration of liposomes and free drug. Cells untreated with any agent are marked as a control.

To check whether the release of cytochrome c from the mitochondria triggers Caspase 9 activation, the Caspase 9 Glo^®^ test was used as in the case of caspase apoptosis (Figure 9). The results confirmed the hypothesis about the activation of the mitochondrial apoptosis pathway in response to the liposomal form of AA and MIT in the presence of vitamin C. The results show an increase in caspase 9 activity 6 hours after administration of liposomes in A375 cells. For the Hs294T line, this increase occurred after the fourth hour of incubation with the liposomes. The results indicate that the apoptosis occurs rapidly. No increase in the activity of this caspase was observed in normal cells, suggesting that another mechanism associated with caspases is responsible for their death.

### 2.5. Tf-Targeted Liposomes Demonstrate Efficient Melanoma Cell Killing

**Figure 10:**
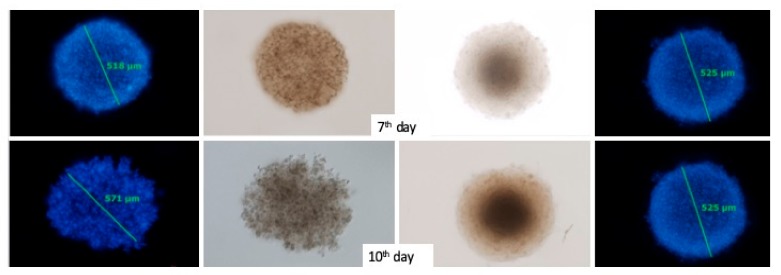
Microscopic image showing spheroids at various growth stages Cell nuclei were stained with Hoechst 33342.

A 3D cell model was used to check how targeted liposomes interact with cells. Cells of melanoma line were plated on agarose plates in varying amounts and cultured for two weeks. As presented in Figure 10, the spheroids were fully mature on the seventh day of culture, as evidenced by the clear boundary of the spheroid. In the following days of the culture, the spheroids gradually changed their shape and the scattering of cells forming the spheroids was observed. Spheroids formed by approximately 13,000 cells on the seventh day of culture with diameter of approximately 500 μm for both cell lines were selected for further experiments.

**Figure 11:**
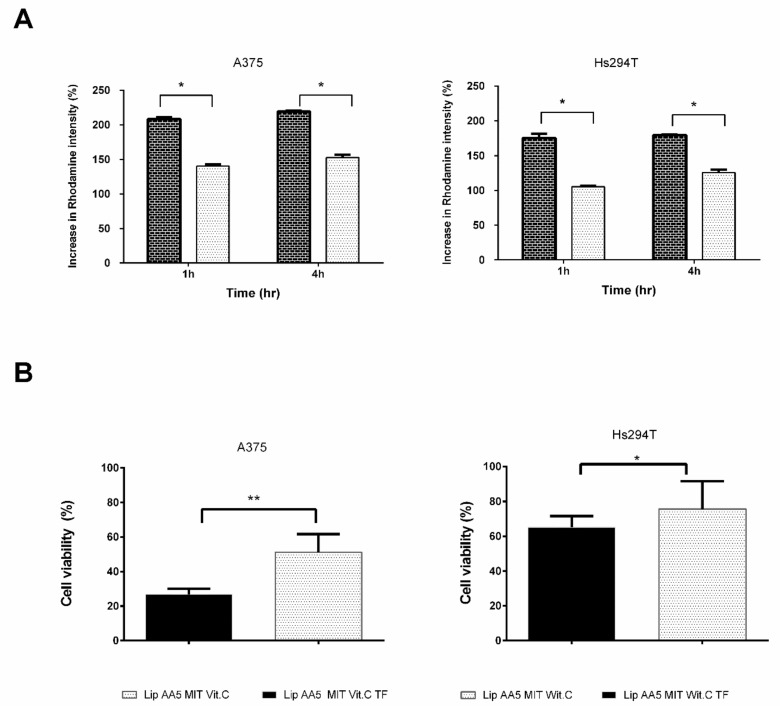
(**A**) Degree of association of liposomes with target A375 and Hs294T cells at 1 and 4 h. Autofluorescence of spheroid forming cells was taken as 100%. The results are the mean ± SD from three independent repetitions. (** two-way ANOVA *p* < 0.01). (**B**) Viability of melanoma cells forming spheroids 72 hours after administration of liposomes. Luminescence of untreated cells was accepted as 100% (control). The results are presented as mean ± SD from five independent repetitions. (test *T* * *p* < 0.05, ** *p* < 0.01).

Transferrin was selected for targeting liposomes. Liposomes were labeled with rhodamine. After incubation, rhodamine fluorescence in the cells was measured by flow cytometry (Figure 11A). Transferrin-targeted liposomes bound to the cells faster than non-targeted liposomes, regardless of the concentration of the liposomes administered. This effect was independent of the cell model. 

The next step was an attempt to achieve greater association efficiency that translates into higher cytotoxicity of liposome formulation. ATP level measurements were performed as a toxicity indicator. The number of viable cells within spheroids, after treatment with liposomes (Figure 11B), indicated that targeting liposomes with transferrin increased the toxic effect of these liposomes. For the A375 cell line, the percent of viable cells that remained after administration of untargeted liposomes was approximately 50%, corresponding to a dose equivalent to the IC_50_. The targeted formulation reduced cell viability by about 75%, so we have a significant increase in efficiency in the action of liposomes. For the second line of melanoma cells, this effect is slightly weaker, however, the difference between the targeted and non-targeted formulations remains statistically significant.

## 3. Discussion

Current chemotherapy is usually insufficient to kill all cancer cells, even at maximum tolerated doses of the cytostatic. Even a relatively small increase in drug resistance in cancer cells may be enough to make a given agent completely ineffective. P-glycoprotein is a protein present on the surface of many types of tumors, and its overexpression is considered one of the main mechanisms by which cancer cells acquire resistance to chemotherapeutics. For this reason, it has been the target of many anticancer therapies. However, drugs such as verapamil and cyclosporine, which inhibit P-glycoprotein have many side effects and their use has become limited [40,41]. Therefore, additional compounds that may inhibit P-glycoprotein have been sought. The present study used a naturally occurring compound that inhibits the function of P-glycoprotein by blocking the NF-kB pathway to sensitize tumor cells to a known chemotherapeutic agent [42]. Anacardic acid is one of those compounds, which has been enriched in the liposomal membrane. The lipid carrier we developed was built from hydrogenated phosphatidylcholine, anacardic acid, dioleylphosphatidyl-ethanolamine, cholesterol, 1,2-distearoyl-sn-glycero-3-phosphatidylethanolamine-N-[amino(poly-ethylene glycol)-2000 and the *para*-nitrophenol derivative DOPE-PEG. In these liposomes mitoxantrone was actively entrapped using the pH gradient generated by ammonium sulfate or ammonium ascorbate.

However, it is known that for anti-cancer therapy to be fully effective, it is necessary to selectively target cancer while maintaining low toxicity towards normal cells. A breakthrough in the treatment of cancer was the transition from non-specific to strictly targeted therapy. The developed carrier represents this by targeting cells overexpressing the receptor for transferrin. The carrier is a targeted liposomal “cocktail” of three active substances with anticancer activity. The carrier of anti-cancer drugs is designed to not only improve its effectiveness, but to also reduce the side effects of the chemotherapeutic agent. In the case of mitoxantrone, its strongest side effects are cardiotoxicity and hepatotoxicity. As we managed to show, the carrier developed, allowed reduction of toxicity to the free drug for Hep-G2 cells. In addition, it was shown that anacardic acid, present in the formulation at 5–40 mole%, did not increase toxicity to liver cells, even in combination with mitoxantrone. A similar effect was obtained for H9C2 myocardial cells, with the exception of the formulation containing 40 mol% AA and MIT and the MIT formulation with AS, which proved to be more toxic than the free drug (Figure 2 and Figure 3). The higher toxicity of the latter formulation suggests the involvement of vitamin c in the protection of cells against toxicity of the drug. The majority of the physiological and biochemical mechanisms of vitamin c’s action result from the fact that it is an electron donor. Vitamin c is a powerful antioxidant due to the fact that, being such a donor, it protects other cellular components against oxidation [43]. Anacardic acid can also have a protective effect on myocardial cells. It allows for the differentiation of relevant stem cells towards cardiomyocytes, by affecting the change of chromatin structure and activation of the Castor 1 transcription factor necessary for the differentiation and maturation of these cells [44].

Next, how the liposomes developed interact with target cells was investigated. In vitro cytotoxicity measurements using melanoma cell lines A375 and Hs294T showed that anacardic acid significantly increases the activity of liposomal mitoxantrone at all concentrations tested, regardless of the gradient generator. One explanation may be the improvement of intracellular drug delivery. It should be noted that AA liposomes without MIT showed only a slight inhibition of melanoma cell line proliferation. As confirmed in these studies, mitoxantrone and anacardic acid act synergistically on melanoma cells in the presence of vitamin C or ammonium sulfate. The combination factor values indicate a very strong effect of this combination (Table 3). It may be more interesting that a combination of the antitumor factor with vitamin C usually causes the reverse effect [23] suggesting a key role for anacardic acid in this combination. Anacardic acid has antitumor activity that can induce apoptosis independent of caspase [45] and inhibit the NF-κB pathway [33]. It is also an epigenetic factor that inhibits the activity of histone acetyltransferases (HAT) involved in the regulation of gene expression. One of the HAT family members, Tip60, participates in cell responses to genotoxic events, such as plexus interruptions [46,47]. Inhibition of this enzyme causes weakening of DNA repair systems. Anacardic acid can sensitize cancer cells to genotoxic damage by inhibiting Tip60 [48]. It is known that one of the main mechanisms of action of mitoxantrone is promotion of DNA damage. Therefore, damage to the repair system by anacardic acid may contribute to the observed increase in toxicity.

An interesting observation is that the cytotoxicity of mitoxantrone and anacardic increased with ascorbic acid compared to liposomes with ammonium sulphate. Free vitamin c has previously been used to enhance the activity of several anthracyclines, in particular doxorubicin (DOX), which led to increased DNA damage caused by ROS [49]. Mitoxantrone and ammonium ascorbate cytoxicity might be due to inhibition of COX expression [50] or activation of the p53 protein by vitamin c with induction of apoptosis, which has been demonstrated with the A375 melanoma cell line [51].

A different effect of this formulation seems very important with respect to normal skin cells, where the value of the combination factor calculated from the MIT survival curves indicates the antagonistic activity of anacardic acid and mitoxantrone in the presence of vitamin c, while in the presence of ammonium sulphate AA, maintains the synergistic effect of these factors. This is most likely due to the fact that ascorbic acid plays a role in protecting cells against genotoxic damage, but the exact protection mechanism is still unclear [52]. The main mechanism of the protective effect of ascorbic acid is its role in ROS removal [53]. However, most likely it is not the main factor that contributes to the protection of NHDF cells against mitoxantrone. Recent profile studies of gene expression in human skin cells treated with ascorbic acid have shown that this effect resulted in an increase in gene expression involved in cell proliferation and DNA repair [54]. Other studies have shown that vitamin c can stimulate chemical repair of DNA [55]. This may partly explain the ROS-independent protective mechanism of ascorbic acid in NHDF cells. It has turned out that liposomal formulations containing anacardic acid do not fully meet the objectives of this project, if the amount of anacardic acid exceeds 15 mol%. This observation is strongly supported by the human red blood hemolysis results. A study has been described previously by Stasiuk et al. the hemolytic activity of anacardic acid towards sheep erythrocytes [56]. As expected, the lytic potential of AA was decreased after its incorporation in liposomes. A similar phenomenon regarding the reduction of hemolytic activity after encapsulation in nanocarriers has already been reported [57,58]. Therefore, formulations containing only 5 mol% were used for the study, by meeting the criteria of stability. This particular formulation was chosen because, in addition to adequate stability, it was characterized by high toxicity to melanoma cells with little toxicity to normal cells. The toxic action of the designed carrier model involved the induction of cancer cells into the ROS-dependent apoptosis pathway. The results of in vitro analyses indicate that the developed lipid carrier effectively delivers the drug to cancer cells, translates into an enhanced production of reactive oxygen species (Figure 5), which then leads to the induction of the apoptosis shown by cytometric analyzes (Figure 6). The mechanism of this phenomenon is known to be dependent on the balance between the expression of pro-apoptotic and anti-apoptotic BCL2 proteins [59,60]. The increase in Bcl-2 levels in cancer cells blocks the release of cytochrome C from the mitochondria. Cytochrome C in the cytosol activates caspase 9 and then 3, leading to apoptosis.

The results suggest that the developed formulation causes the entry of tumor cells into the mitochondrial-dependent apoptosis pathway, as evidenced by the activity of caspase 9 (Figure 9). They also suggest a different mechanism of death for normal and cancerous cells. Based on the activity of caspases, a potential mechanism of action of the developed liposomes is proposed.

The proposed apoptosis model is based mainly on the lack of caspase 9 activation in NHDF cells and activation of executive caspases of apoptosis in all cellular models (Figure 7). The higher efficiency in introducing cells to the apoptosis pathway, and thus higher cytotoxicity, is dependent on the amount of drug delivered to the cells. The new mitoxantrone vehicle was designed so that the efficiency of active encapsulation was very high (over 95%) and the carrier was stable. However, it has been reported that even active encapsulation of the drug causes very rapid clearance of liposomes from the bloodstream (less than 5% of the drug remained in the bloodstream for more than two hours) [61]. In the case of sterically stabilized liposomes containing the PEG-lipid conjugate, over 80% of lipids and drug were present in the blood 4 hours after injection, and after 24 hours it remained more than 30%. Unfortunately, even prolonged circulation in the bloodstream does not necessarily lead to an increase in the effectiveness of the drug. It is believed that sterically stabilized liposomes containing mitoxantrone do not release the drug inside the cells. Instead there is a simple fusion of the liposome with the cell membrane and passive drug passage into the cell, or the drug is released as a result of carrier metabolism by macrophages present in tumor tissue [62,63]. One of the methods to reduce this effect is by targeting liposomes. In turn, longer persistence of liposomes in the blood enables better action on circulating cancer cells, which allows elimination of cells that metastasize to other organs [64]. In the case of melanoma, this is especially important because the primary way of treating skin cancers is surgical excision of the lesion and therapy preventing metastases. This argument supports the use of liposomes as a drug carrier in melanoma therapy.

As mentioned before, a desirable carrier feature for anti-cancer therapy is a high specificity that allows the drug to be introduced into the cells in a targeted and controlled manner. The developed liposomes with anacardic acid, containing ammonium ascorbate and mitoxantrone, may increase drug delivery to cells, by prolonging circulation time and targeting tumor tissue through the EPR effect, which is a known property of liposomes. 

A very important step in the proposed project was to test the lipid carrier in the presence of transferrin. The addition of DOPE-PEG3400-pNP and the attachment of ligand to the surface of liposomes did not cause significant changes in the basic physicochemical properties of the preparation. The presence of DOPE in the coating promotes the fusion of liposomes with the endosomal membrane [65,66], which has been considered the most crucial step for the effective action of anticancer drugs [65]. In this case DOPE’s main effect was to anchor the liposome of the long-chain PEG associated with the targeting factor. Using confocal microscopy and flow cytometry techniques, we observed clear differences in rhodamine-labelled Lip AA5 MIT Vit. C TF liposome effects with membrane of cells of different melanoma lines compared with Lip AA5 MIT Vit.C (Figure 11A). High specificity of interaction of the designed carrier with cells displaying the receptor for transferrin was also demonstrated. Furthermore, the results of cytotoxicity analysis (Figure 11B) indicate the relatively high specificity of TF immunoliposomes for target cells. The increased cytotoxicity of transferrin-targeted liposomes may be because these liposomes simultaneously delivered iron and vitamin c to cells. As a result, the iron pool available for the Fenton reaction doubles, since the vitamin c receives iron from ferritin, making them bioavailable [5,67].

High efficacy allows the potential use of this formulation in the therapy of other cancers, since transferrin receptor is overproduced in many types of cancer, especially those that show high growth rates [68,69,70]. The use of transferrin as a targeting agent is common, both for the management of anti-cancer drugs alone [71,72,73], for proteins with toxic effects [74,75,76] and for nucleic acids [77,78].

The targeting of the first type of transferrin receptor (TFR1) is made possible, by strategies that utilize its natural TF ligand, monoclonal antibodies or their fragments. Unfortunately, the blocking of the cytotoxicity of TF conjugates by native TF is a disadvantage. Therefore, its high concentrations circulating for long periods may disrupt the action of these conjugates, leading to a decrease in their therapeutic effectiveness. Because TF conjugates have the potential to interact with both TFR1 and TFR2 (which are predominantly present in the liver), they can be toxic to many normal cells. Targeting the TFR1 receptor carrier using monoclonal antibodies can help bypass these potential problems [79]. 

Hypoxia is a common feature of solid tumors and associated with tumor progression [80,81]. The expression of TFR in cells in an anaerobic microenvironment may be a more rational and effective use with targeted TFR molecules. The results of the tests conducted with the A375 cell line in hypoxia showed an increased expression of TFR in hypoxia at both the mRNA and protein level, which was confirmed by surface TFR analysis measured by flow cytometry [82]. The results of these tests confirm the validity of our use of transferrin as a driving factor, since its expression depends on the tumor environment. The developed liposomal formulation is a promising anticancer agent that may also be used as the basic platform for further modification.

In vitro studies have their limitations that make the interpretation of results difficult and do not always reflect the response of cancer cells growing in vivo. This is because most commercially available cancer lines have been derived by performing serial passages and selection of cells with desired traits, such as the expression of specific genes, morphological traits and functions. During their controlled growth, cancer cells acquire phenotypic traits that allow them to adapt to in vitro conditions [83]. In addition, in unilamellar cultures, the cells are provided with easy access to nutrients and oxygen, resulting in a homogeneous genotype and phenotypic cell population [84]. It should be emphasized that tumor cells cultured under such conditions lack the complexity of a tumor structure that grows in vivo [85]. Therefore, to evaluate the effectiveness of the developed anti-cancer drug carrier, spheroids (three-dimensional cultures)—aggregates of tumor cells that are grown in vitro were used. Multicellular tumor spheroids exhibit tumor traits that occur in vivo in the early phase of their growth and are therefore considered to be an intermediate form between cells from monolayer type cultures and spontaneously growing tumors [86,87,88]. 

## 4. Materials and Methods

### 4.1. Materials

Hydrogenated soy phosphatidylcholine (HSPC), 1,2-distearoyl-sn-glycero-phospho-ethanolamine-N-[poly(ethylene glycol)2000] (DSPE-PEG2000) were from Lipoid GmbH (Ludwigshafen, Germany). Cholesterol (CHOL) and 1,2-dioleoyl-sn-glycero-3-phosphoethanolamine (DOPE) were purchased from Northern Lipids, Inc. (Vancouver, BC, Canada). Nitrophenyl carbonate-PEG-nitrophenyl carbonate, MW 3400 (pNp-PEG-pNp) was purchased from Laysan Bio, Inc. (Arab, AL, USA). Natural cashew nutshell liquid (CNSL) was from Sandor Cashew (Delhi, India). AA was extracted from natural CNSL and subsequently hydrogenated using a method described by Legut et al. [89]. Sodium dihydrogen phosphate, disodium hydrogen phosphate, sodium chloride, dimethyl sulfoxide (DMSO), hydrogen peroxide, ascorbic acid and ammonium hydroxide were purchased from POCH (Gliwice, Poland). Sephadex G-50 Fine, Sepharose CL-4B, 3-(4,5-dimethylthiazol-2-yl)-2,5-diphenyltetrazolium bromide (MTT), rhodamine B, triethylamine (TEA), molybdenum blue spray solution, Dragendorff spray reagent, ninhydrin spray solution, transferrin, pyruvate, anti-cytochrome C antibody (N-terminal), paraformaldehyde (PFA), protease inhibitor cocktails and 4-Nonylphenyl-polyethylene glycol (Nonidet^TM^ P40) where obtained from Sigma-Aldrich (St. Louis, MO, USA). Mitoxantrone hydrochloride (MIT) was a gift from the Pharmaceutical Research Institute (Warsaw, Poland). Cell culture media (DMEM, MEMα,), antibiotic-antimycotic solution and DPBS buffer were purchased from Lonza (Basel, Switzerland). Fetal Bovine Serum (FBS) was purchased from EuroClone (Pero, Italy). Nucleopore^TM^, filters were obtained from WHATMAN^®^ (Maidstone, UK). Hoechst 33342, GlutaMAX, 2′, 7′ DCFH-DA (2′, 7′-dichlorodihydrofluorescein diacetate); MitoTracker^®^ Red CMXRos were purchased from Life Technologies (Carlsbad, CA, USA). Annexin V Apoptosis Detection Kit was obtained from BD Pharmingen™ (Franklin Lakes, NJ, USA). CellTiter-Glo^®^ Luminescent Cell Viability Assay, Caspase-Glo^®^ 3/7 Assay, Caspase-Glo^®^ 9 Assay and GSH-Glo™ Glutathione Assay were purchased from Promega (Madison, WI, USA). All the other reagents were of analytical grade.

### 4.2. Preparation and Characterization OF Liposomes

Liposomes were prepared by a lipid hydration, freeze-and-thaw (FAT) procedure and extrusion through polycarbonate filters as described before [89]. In brief chloroform stock solutions of lipids (HSPC, CHOL, DSPE-PEG2000 and AA) were prepared and appropriate volumes were then used to obtain specific molar ratios of lipids presented in Table 4.

Chloroform solutions of the lipids were mixed, and chloroform was then evaporated using a nitrogen stream. Thin lipid films were dissolved in cyclohexane and the mixture subsequently frozen in liquid nitrogen and freeze-dried overnight at low pressure using a Savant Modulyo apparatus (Savant, Waltham, MA, USA). 

Thin lipid films were hydrated with 300 mM ammonium ascorbate, pH = 4.0 or 300 mM ammonium sulfate, pH 5.5. The obtained multilamellar vesicles (MLVs) were freezed and thawed (FAT) ten times, followed by extrusion 10 times through polycarbonate filters with pore size of 100 nm on Thermobarrel Extruder (PPHU Marker, Wroclaw, Poland). 

The ion and/or pH gradient was subsequently generated by exchanging the extravesicular liposomal solution on Sephadex G-50 Fine (1 × 20 cm) columns as described previously [89].

Afterwards liposome size, polydispersity index (PDI) and zeta potential were determined by the dynamic light scattering, Zetasizer Nano-ZS (Malvern Instruments Ltd., Malvern, UK). Phospholipid concentration was determinated by ammonium ferrothiocyanate assay [90] on the Shimadzu UV 2401 PC spectrophotometer (Shimadzu, Kyoto, Japan). Methanolic solution of liposomes was used for mitoxantrone concentration measurement at λ = 667 nm.

After the remote loading, non-encapsulated drug was separated from MIT-containing liposomes on Sephadex G-50 Fine mini-columns (5.5 × 70 mm) and the MIT in collected fractions was determined. The encapsulation efficiency (EE) was calculated according to the formula: EE [%] = ((MIT[mM])/(lipid [mM]))/(initial D⁄(L)), where initial D/L is the drug-to-lipid ratio when drug was mixed with liposomes. The morphology of liposomes was determined by using a TESLA BS 540 transmission electron microscope. A drop of liposomes was placed on a copper grid and dried at room temperature. This step was followed by staining with 2% uranyl acetate and dried at room temperature. 

### 4.3. Targeting of the Liposomes

#### 4.3.1. Synthesis of pNP-PEG-PE

The pNP-PEG-PE synthesis reaction was carried out at room temperature by mixing pNP-PEG-pNP dissolved in chloroform and DOPE in the ratio of 4.8:1 as described [91]. The progress of the reaction was monitored by TLC chromatography. Dragendorff reagent was used to visualize PEG, molybdenum blue to visualize phosphate groups and ninhydrin to visualize primary amino groups. After completion of the reaction, the solvent was evaporated. The next step purified the product by gel filtration (Sepharsose CL-4B (2.5 × 50 cm). To identify the fractions containing the product, thin layer chromatography and visualization identical to that used during reaction monitoring were used. The fractions containing the product were frozen and lyophilized. The resulting and purified product was used for further modifications.

#### 4.3.2. Attachment of Protein

The pNP-PEG-PE conjugate was used for the following reaction. A thin lipid film of the conjugate was hydrated with the transferrin saline solution (4 × molar excess to pNP-PEG-PE). The spontaneous hydrolysis of *para*-nitrophenol and the attachment of amino groups from peptide to terminal PEG chains were allowed for 12 hours at 4 °C. The excess of unbound transferrin was removed by dialysis into normal saline. The reaction efficiency was then checked by measuring protein with the BCA test. To obtain a targeted formulation, Tf-PEG-PE micelles were added to pre-prepared liposomes for post-insertion of PEG with attached targeting moieties. The insertion process was for 12 hours at 37 °C with gentle agitation. 

### 4.4. Ex Vivo Red Blood Cell Hemolysis Assay

The study was approved by the Bioethics Commission at the Lower Silesian Medical Chamber (1/PNHAB/2018). Hemolysis was determined by measurement of hemoglobin release from human erythrocyte suspensions after incubation with a methanolic solution of AA or different liposomes formulations as previously described [92].

### 4.5. Cell Culture and Treatment

Human melanoma cell lines A375 (kindly provided by the Laboratory of Cell Pathology, Faculty of Biotechnology, University of Wroclaw, Poland) and Hs294T (kindly provided by Institute of Immunology and Experimental Therapy, Polish Academy of Sciences, Wroclaw, Poland) was cultured in DMEM medium supplemented with 2 mM glutamine and 5 or 10% fetal bovine serum. The human skin fibroblast cell line NHDF (CC-2511, Lonza) was cultured in MEM α medium supplemented with 2 mM glutamine and 10% fetal bovine serum. All the media contained 100 U/mL penicillin, 0.1 mg/mL streptomycin and 0.25 µg/mL amphotericin B. Ninety-six-well plates coated with 1.5% agarose were used to obtain the spheroids. 12,000 cells were plated on such prepared plates and the plates were centrifuged for 15 minutes at 10 °C at 3000 rpm. The cells were cultured at 37 °C in humid atmosphere saturated with 5% CO_2_.

### 4.6. Cell Viability

#### 4.6.1. MTT Assay

The cell viability was determined using a MTT assay by the method described by Mosmann [18]. Briefly, 10,000 cells/well were seeded onto 96-well plates and allowed to adhere for 24 hours. After treatment with liposomes and free drug cells were incubated in a solution of MTT in culture medium (0.5 mg/mL) for 4 hours. After incubation, the solution was removed and 0.05 mL of DMSO was added to each well, and plates were shaken gently for 5 min. Absorbance was measured at 550 nm with a reference wavelength of 630 nm on a UVM 340 microplate reader (Biogenet, Jozefow, Poland). The cell viability was estimated as the percentage of the control, which was cells untreated with any agents (100%). All experiments were performed a minimum of three times in triplicate.

#### 4.6.2. ATP Measurement

A CellTiter-Glo^®^ kit was used to measure cell viability. Cells were plated in a 96-well white plate at 10,000 cells/well and then incubated with the liposomes for a specified time. After incubation, the medium was replaced by fresh medium (100 μL). The plate was then left for 30 minutes at room temperature. An equal volume of CellTiter Glo reagent was added and incubated for 2 minutes with shaking to lyse the cells. Luminescence measurements were made using an EG & G Berthold luminometer (Wildbad, Germany).

### 4.7. Determination of the Association of Targeted Liposomes with Cells

After treatment with rhodamine-labeled liposomes (rhodamine-DOPE was added to the lipids at 1% mol ratio during preparation), spheroids were harvested at specified time intervals and rinsed twice with PBS and then trypsinized to break down the spheroid structure into a single cell suspension. The cells were centrifuged and rinsed again with PBS buffer. The cells were analyzed using a BD FACS Calibur^TM^ (BD Biosciences, San Jose, CA, USA) flow cytometer based on 10,000 counts for each population tested. The fluorescence of rhodamine labeled cells was evaluated using a standard FL-2 filter, λ = 585 nm. The BD CellQuest^TM^Pro software (version 5.2.1, BD Biosciences^®^) was used to analyze and visualize the results. Single samples were represented by 10 randomly selected spheroids from a given group.

### 4.8. Analysis of Apoptosis

Apoptosis and necrosis of treated cells was measured using flow cytometry using a FACSCalibur instrument (BD Biosciences) with a modified version of the protocol described previously [19]. Briefly, cells (500,000 cells/well) were grown on 6-well plates. Thirty minutes before the end of incubation, hydrogen peroxide (10% v/v) was added to the cells for a positive control of apoptosis. As a positive control for necrosis, 8 µL of Triton X-100 (0.1% v/v) was added to the cells and incubated for 5 min at room temperature. After incubation, cells were harvested by trypsinization and centrifuged (2000 rpm, 5 min, room temperature) and processed according to the manufacturer protocol. The results were analyzed using CellQuest^™^ Pro software (BD). The results from treated cells were compared to control, untreated cells. All experiments were performed a minimum of three times in triplicate

### 4.9. Lactate Dehydrogenase Leakage Assay

A lactate dehydrogenase (LDH) leakage assay was used to measure hepatotoxicity. Lactate dehydrogenase activity in the culture medium was used as an indicator of the integrity of the cell membrane. The LDH assay was performed as a modification of the method described previously [20]. Briefly, cells (50,000 cells/well) were grown on 48-well plates. Cells were treated with liposomes and free drug. After the incubation, the culture medium was collected, and the cells were washed twice with PBS. Then, the cells were lysed with 100 µL of lysis buffer Nonidet P-40 consisting of 1% Triton X-100, 150 mM NaCl and 50 mM Tris HCl (pH 8.0) with a protease inhibitor cocktail for 10 min at 4 °C. Lysates were collected and centrifuged (10,000 rpm, 10 min, 4 °C), and the supernatant was collected and stored at 4°C. The activities of LDH in the medium (LDH_out_) and in the cells (LDH_in_) were measured according to the manufacturer’s instructions (Biosystems Reagents & Instruments, Quezon City, Philippines). The enzyme activity was measured at 37 °C via quantification of NADH (1.55 mM) consumption using spectrophotometry (at λ = 340 nm) with pyruvate (2.75 mM) as the substrate in 100 mM Tris-HCl buffer (pH 7.2). The absorption was read directly (T_0_) and after 3 min (T_3_) using a UV-2401PC spectrophotometer (Shimadzu, Kyoto, Japan). The cytotoxicity = LDH_out_/(LDH_out_ + LDH_in_) expressed as a percentage of the control, untreated cells. All experiments were performed a minimum of three time in triplicate.

### 4.10. Measurement of Intracellular Reactive Oxygen Species

Fluorescence spectrophotometry was used to measure the intracellular levels of ROS with 2′,7′-dichlorodihydrofluorescein diacetate (H_2_DCFDA) as the probe. The measurements were conducted by modifying the manufacturer protocol. Briefly 10,000 melanoma cells and control cells were grown in 96-well black plates and treated with liposomes at selected time intervals (at 1 to 24 hours). The cells were treated as described in the MTT assay. Ammonium iron (II) sulfate (Mohr’s salt, 50 mM) was added to the cells as a positive control. After incubation, the culture medium was removed, cells were washed twice in PBS, and 100 µL of probe (10 µM in DPBS) was added to each well and incubated for 30 min at 37 °C in the dark. After incubation, the probe was removed, and the cells were washed twice in PBS. Then 200 µL of DPBS was added to each well and the intensity of fluorescence was read immediately on a Varian Cary Eclipse Fluorescence Spectrophotometer (Varian Ltd., Victoria, Australia) at λ = 498 nm for excitation and at λ = 525 nm for emission. Results were expressed as a percentage of arbitrary units of fluorescence compared to the control level, which was the cells untreated with any agents. All experiments were performed a minimum of three times, in triplicate. 

### 4.11. Caspase 9 and 3/7 Assays

Harvested cells were seeded on white 96-well microplates with 10, 000 cells/well and allowed to adhere overnight and then treated with liposomes. After treatment, plates containing cells were removed from the incubator and allowed to equilibrate to room temperature. Caspase-Glo^®^ 9 Reagent or Caspase-Glo^®^ 3/7 Reagent was added to each well containing 100 μl of blank, negative control cells or treated cells in culture medium. Contents of plates were gently mixed using a plate shaker at 300–500 rpm for 2 minutes and incubated at room temperature for 30 minutes. Chemiluminescence measurement was done with an EG&G Berthold luminometer. The results are expressed as the percentage with respect to the control (stained cells). 

### 4.12. Cytochrome C Staining

Harvested cells were seeded on cover glasses (50,000 cells), allowed to adhere overnight before treatment with liposomes. After treatment, cells were washed with PBS and then fixed and permeabilized. Primary anti-cytochrome C antibodies, secondary antibodies (FITC), Hoechst dye and MitoTracker^®^ Red CMXRos dye were used for staining. Cells were incubated with primary antibodies (1: 100 dilution) for 12 hours at 4 °C, followed by incubation with secondary antibodies (1: 200 dilution) for two hours at room temperature. Incubation with MitoTracker Red^®^ (250 nM) dye lasted 20 minutes at room temperature. For visualization Zeiss LSM 880 Airyscan confocal microscope (Zeiss, Jena, Germany) was used.

### 4.13. Statistical Analysis

Results were presented as mean values and standard deviations. Statistical significance analysis of data differences was performed using the GraphPad Prism software (software version 7.0 VA, GraphPad Software, San Diego, CA, USA). The IC_50_ value was also determined using that software. Combination index values (CI) were calculated using CompuSyn software version 1.0 (freeware, The ComboSyn, Inc., Paramus, NJ, USA). 

## 5. Conclusions

Here, we have prepared transferrin targeted liposomal formulations for co-delivery of mitoxantrone, anacardic acid and ammonium ascorbate to melanoma cells. We have demonstrated that one of developed liposomal formulations enhanced the level of apoptosis and cell death in melanoma cell lines but not in normal cells. The proposed mechanism of the cytotoxic action of liposomes involves specific generation of the free radicals by an iron ions mechanism and the induction of the apoptotic pathway in the melanoma cells. In summary we have successfully prepared a peptide-modified co-delivery system for chemotherapeutics and natural bioactive substances with an enhanced active tumor-targeting effect and therapeutic efficacy. 

## Figures and Tables

**Table 1:** Summary of size (Z-average), polydispersity index (PDI) and zeta potential of (**A**) all nano-preparations and (**B**) 5mol% anacardic acid nano-preparations before and after targeting with transferrin.

**A**				
**Amount of Anacardic Acid in Liposome Membrane (mol%)**	**Diameter** **(nm)**	**Zeta Potential (mV)**	**PDI**	**Encapsulation Efficiency (%)**
**0**	112 ± 1.2	0.87 ± 0.39	0.036	99.5
**5**	111 ± 1.5	−4.31 ± 0.49	0.042	99.6
**10**	115 ± 2.3	−5.42 ± 0.63	0.051	98.9
**15**	112 ± 0.8	−5.74 ± 1.1	0.046	99.2
**20**	105 ± 2.5	−4.12 ± 0.9	0.032	99.2
**40**	110 ± 2.1	−2.81 ± 0.5	0.06	98.6
**B**				
**Liposomal Formulation**	**Diameter** **(nm)**	**Zeta Potential (mV)**	**PDI**	**Encapsulation Efficiency (%)**
**LipAA5 Vit.** C	111 ± 1.5	−4.31 ± 0.49	0.042	99.0
**LipAA5 Vit. C TF**	119 ± 1.5	−3.71 ± 0.5	0.05	89.9

**Table 2:** Comparison of IC_50_ values (μM) of mitoxantrone-free liposomes and mitoxantrone-containing liposomes for all cell lines. Values are averages of three independent measurements with standard deviation.

Liposomal Formulation	Cell Line					
	A375		Hs294T		NHDF	
	IC_50_ 48 h	IC_50_ 72 h	IC_50_ 48 h	IC_50_ 72 h	IC_50_ 48 h	IC_50_ 72 h
Lip Vit.C	286.5 ± 31	158.37 ± 35	74.92 ± 7.5	43.95 ± 0.3	174.3 ± 7.3	122.06 ± 9
LipAA5 Vit.C	15.4 ± 3	12.63 ± 5	30.43 ± 6.65	0.52 ± 0.03	188.5 ± 20.8	152.93 ± 11
Lip AA10 Vit.C	17 ± 0.3	58.71 ± 7.4	3.54 ± 0.5	1 ± 0.03	23.4 ± 1.5	20.01 ± 0.46
Lip AA15 Vit.C	14.8 ± 0.08	1.78 ± 0.5	1.93 ± 0.5	5.59 ± 1.34	19.57 ± 0.8	19.32 ± 0.93
Lip AA20 Vit.C	17.7 ± 2.1	2.71 ± 0.55	1.7 ± 0.4	0.6 ± 0.04	26.87 ± 3	12.12 ± 2.8
Lip AA40 Vit.C	1.35 ± 0.2	0.93 ± 0.09	1.26 ± 0.07	0.29 ± 0.06	20 ± 1.3	9.1 ± 0.84
Lip AS	240.5 ± 57	99.06 ± 14.2	123.15 ± 7	86.33 ± 6.8	44.5 ± 3.6	6 ± 1.1
Lip AA5 AS	36.1 ± 6	92.37 ± 18.9	109.43 ± 7.7	55.64 ± 9.4	58.36 ± 6.1	5.4 ± 1.3
Lip MIT Vit.C	50.44 ± 4.3	10.58 ± 3	19.52 ± 7.6	10.3 ± 2	41.25 ± 3.21	20.48 ± 4.25
Lip AA5 MIT Vit.C	0.4 ± 0.05	0.24 ± 0.1	1.69 ± 0.3	0.66 ± 0.13	84.04 ± 12.7	44.19 ± 5.7
Lip AA10 MIT Vit.C	0.47 ± 0.02	0.39 ± 0.02	1.88 ± 0.33	0.94 ± 0.04	4.7 ± 0.43	1. ± 0.04
Lip AA15 MIT Vit.C	0.37 ± 0.07	0.39 ± 0.01	0.77 ± 0.05	0.18 ± 0.005	1.5 ± 0.28	0.9 ± 0.006
Lip AA20 MIT Vit.C	0.46 ± 0.03	0.28 ± 0.002	0.18 ± 0.001	0.1 ± 0.007	0.84 ± 0.03	0.23 ± 0.009
Lip AA40 MIT Vit.C	0.04 ± 0.003	0.02 ± 0.002	0.12 ± 0.06	0.07 ± 0.006	0.3 ± 0.02	0.19 ± 0.004
Lip MIT AS	3.87 ± 1.34	32.5 ± 2.51	17.71 ± 1.22	7.11 ± 1.42	40.53 ± 6.2	1.03 ± 0.06
Lip AA5 MIT AS	19.88 ± 4.15	0.6 ± 0.2	16.6 ± 1.7	1.19 ± 0.26	35.92 ± 3.15	1 ± 0.28
MIT	0.22 ± 0.014	0.075 ± 0.001	0.165 ± 0.03	0.13 ± 0.02	0.43 ± 0.02	0.15 ± 0.02

**Table 3:** Combination index (CI) values of liposomes containing mitoxantrone and anacardic acid in the presence of ammonium sulphate or ammonium ascorbate The CI values are at MIT concentration (50 μM).

Liposomal Formulation	Cell Line		
	A375	Hs294T	NHDF
Lip AA5 MIT AS	0.011	0.085	0.366
Lip AA5 MIT Vit.C	<0.01	0.194	57.064

**Table 4:** The table presents a full list of the formulations developed and their lipid composition along with abbreviations. The last column contains the name of the factor used to generate the transmembrane pH gradient in liposomes.

Formulation Name/Abbreviation	HSPC	AA	DSPE-PEG2000	Cholesterol	Drug/Lipid Ratio	Ion Gradient Generator
Lip MIT Vit. C	55	0	5	40	0.2	Ammonium ascorbate
Lip AA5 MIT Vit. C	55	5	5	35	0.2	Ammonium ascorbate
Lip AA10 MIT Vit. C	55	10	5	30	0.2	Ammonium ascorbate
Lip AA15 MIT Vit. C	55	15	5	25	0.2	Ammonium ascorbate
Lip AA20 MIT Vit. C	55	20	5	20	0.2	Ammonium ascorbate
Lip AA40 MIT Vit. C	55	40	5	0	0.2	Ammonium ascorbate
Lip AA5 Vit. C	55	5	5	35	0	Ammonium ascorbate
Lip AA10 Vit. C	55	10	5	30	0	Ammonium ascorbate
Lip AA15 Vit. C	55	15	5	25	0	Ammonium ascorbate
Lip AA20 Vit. C	55	20	5	20	0	Ammonium ascorbate
Lip AA40 Vit. C	55	40	5	0	0	Ammonium ascorbate
Lip AS	55	0	5	40	0	Ammonium sulfate
Lip MIT AS	55	0	5	40	0.2	Ammonium sulfate
Lip AA5 AS	55	5	5	35	0	Ammonium sulfate
Lip AA5 MIT AS	55	5	5	35	0.2	Ammonium sulfate

AA—anacardic acid.
